# Sensing Urban Patterns with Antenna Mappings: The Case of Santiago, Chile †

**DOI:** 10.3390/s16071098

**Published:** 2016-07-15

**Authors:** Eduardo Graells-Garrido, Oscar Peredo, José García

**Affiliations:** 1 Data Science Institute; Faculty of Engineering, Universidad del Desarrollo, Las Condes 7610658, Chile; 2 Telefónica I+D; Av. Manuel Montt 1404, Third Floor, Providencia, Providencia 7501105, Chile; oscar.peredoa@telefonica.com (O.P.); joseantonio.garcia@telefonica.com (J.G.)

**Keywords:** Call Detail Records, urban dynamics, human mobility, origin-destiny matrix, land use clustering, crowdsourced data, OpenStreetMap

## Abstract

Mobile data has allowed us to sense urban dynamics at scales and granularities not known before, helping urban planners to cope with urban growth. A frequently used kind of dataset are Call Detail Records (CDR), used by telecommunication operators for billing purposes. Being an already extracted and processed dataset, it is inexpensive and reliable. A common assumption with respect to geography when working with CDR data is that the position of a device is the same as the Base Transceiver Station (BTS) it is connected to. Because the city is divided into a square grid, or by coverage zones approximated by Voronoi tessellations, CDR network events are assigned to corresponding areas according to BTS position. This geolocation may suffer from non negligible error in almost all cases. In this paper we propose “Antenna Virtual Placement” (AVP), a method to geolocate mobile devices according to their connections to BTS, based on decoupling antennas from its corresponding BTS according to its physical configuration (height, downtilt, and azimuth). We use AVP applied to CDR data as input for two different tasks: first, from an individual perspective, what places are meaningful for them? And second, from a global perspective, how to cluster city areas to understand land use using floating population flows? For both tasks we propose methods that complement or improve prior work in the literature. Our proposed methods are simple, yet not trivial, and work with daily CDR data from the biggest telecommunication operator in Chile. We evaluate them in Santiago, the capital of Chile, with data from working days from June 2015. We find that: (1) AVP improves city coverage of CDR data by geolocating devices to more city areas than using standard methods; (2) we find important places (home and work) for a 10% of the sample using just daily information, and recreate the population distribution as well as commuting trips; (3) the daily rhythms of floating population allow to cluster areas of the city, and explain them from a land use perspective by finding signature points of interest from crowdsourced geographical information. These results have implications for the design of applications based on CDR data like recommendation of places and routes, retail store placement, and estimation of transport effects from pollution alerts.

## 1. Introduction

Some cities are growing faster than their own ability to adapt to change. This is particularly true in growing economies from developing countries, where population tends to concentrate in their capitals, and urban planners are not prepared for the unexpected growth nor for the mobility required by the population to have a good quality of life.

Urban policy is designed having several inputs in consideration. Travel surveys are one of them, because they provide rich information about the city: where people travel to (and from), the purpose of the trip (e.g., commuting to work), the mode (e.g., metro), the time spent traveling, as well as other socio-demographic variables. However, surveys are expensive, take enormous time and effort to be collected, and may have sampling biases or reporting errors [1,2]. They represent static pictures of a dynamic phenomena and, due to its sample size, they are limited to big areas (either administrative or designed). Yet, in spite of these limitations, travel surveys provide understanding of general urban patterns. But, as expected due to their characteristics, they can not match the speed of urban growth, and thus, subsequent surveys only capture the big patterns and their changes. Motivated by those shortcomings, we propose to use mobile data to analyze and understand urban dynamics. Concretely, we focus on *Call Detail Records* (CDR), data logs used by telecommunications companies to bill consumers. As seen on the literature, CDR data can also be used to sense human mobility [3,4,5,6]. This has potential to help urban planners and policy designers because CDR can be obtained effortlessly, and with volumes that allow a greater granularity of analysis (e.g., smaller city areas) with specific time windows of information collection (e.g., particular days or weeks of data instead of the many months needed for a travel survey).

A common assumption with respect to geography when working with CDR data is that the position of a device is the same as the Base Transceiver Station (BTS) it is connected to. Because the city is divided into a square grid, or by coverage zones approximated by Voronoi tessellations, CDR network events are assigned to corresponding areas according to BTS position. This geolocation may suffer from non negligible error in almost all cases, introducing error in any geographical study being made. When antenna density is high this may not be a problem, but in city areas with lower antenna densities it may need some care. However, on the literature there are two typical geographical units to study CDR data. On the one hand, the city is divided in a regular grid, losing important land use information as all areas are equally sized. On the other hand, the city it is divided mathematically according to antenna coverage. Even though antenna density is correlated with network usage (and thus, floating population), these zonings do not respect natural (e.g., rivers) nor administrative borders (e.g., streets, highways, train lines, etc.).

Having our motivation into consideration, the following are the contributions of this paper:

1. We present “Antenna Virtual Placement” (AVP), a method to geolocate mobile devices connected to network antennas, based on the technology and orientation of the antenna, and a post-processing using Voronoi tessellation. This method decouples the antennas from the cell towers, which is the common spatial unit in the literature.
2. We present a method to estimate two important places for a person using a mobile device: *home* and *work*. This method works reliably with the information of one day, and has potential to improve accuracy by considering more days.
3. We present a method to cluster areas of the city based on floating population patterns measured through mobile connectivity. We use crowdsourced information to explain and characterize those clusters according to land use.

To evaluate our proposed methods we perform a case study using an anonymized CDR dataset from the largest telecommunications company in Chile, with a market share of 38.18% as of June 2015. Chile is one of the developing countries with highest mobile phone penetration—there are 132 mobile subscriptions per 100 inhabitants, implying that the number of subscriptions is greater than the population [7]. We focus our analysis on Santiago, its capital and most populated city. Santiago has experienced accelerated growth during the last decades, a trend that has been predicted to continue at least until 2045 [8].

Finally, we discuss the implications of these contributions for the development of urban computing applications [9], as well as limitations and future work.

## 2. Background

When using mobile data, the core datum is what is called a *network event* [10]. A network event indicates when a mobile device has connected to an antenna from the mobile network. Available connections include calls, text and multimedia messages, as well as Internet events. The regularity of these events may differ: sometimes every connection is available, sometimes only the billable ones are. Calls and messages are always billable, but Internet connections are not—the number of packages sent through the antennas may be very high, but billing is performed according to the number of megabytes transmitted. Thus, based on those events, it is possible to build spatio-temporal trajectories based on the transitions between antenna connections performed by mobile devices.

However, aggregated transitions require a well defined zoning of the city. Usually, these zones are built around cell towers or *Base Transceiver Stations* (BTS), considering Voronoi diagrams that approximate the coverage areas of the antennas within each BTS [11,12,13]. We apply a different approach, because we work with designed zonings (like [10]). This is a desirable approach because working with designed zonings allows comparison with travel surveys. However, in this scenario, BTS’ geographical coordinates to locate mobile devices does not reflect their true position. Previous work to obtain those positions include the usage of probabilistic simulations of each device position, by estimating an a priori cumulative density function (CDF) on the device location related with the corresponding BTS [14]. This approach requires a parameter which controls the speed of signal decay, and must be inferred from field GPS data in each zone of interest. If real-time signal strength with nearby antennas is available, then high resolution mappings can be obtained [15], but this is information is not always available due to the associated technological costs. Thus, our proposal is based on a decoupling of the antennas on each BTS to estimate a likely position for the mobile devices connected to it. We do so by employing Voronoi tessellations with an increased number of sample points of artificial positions for each antenna within a BTS.

Because CDR data exposes movement traces, it is possible to estimate important places for a device under the assumption that devices represent individuals [16,17,18,19,20]. Such important places are usually understood as *home* and *work*; other places may fall in a wide range of venues, known as *third places*. A characteristic of home and work is that individuals spend most of their time in them, and they do so frequently. Several methods have been tested to identify those places based on spatio-temporal patterns: clustering [18,20], conditional random fields [19], and spatial modeling (e.g., standard deviational ellipse) to build anchor-point models [16,17]. They do not work only with mobile data from phone operators, but also with other sources like WiFi signals [18] and GPS traces [19]. In those works, the mobile traces span from several days to months. Conversely, in this paper we propose to detect these meaningful places using a single day of mobile traces allows us to predict home and work, using probability distribution fitting and time window weighting. Since one of the primary outcomes of travel surveys are the so-called Origin-Destiny (OD) matrices, which have also been studied using mobile data [10,11,12,13], we evaluate our results by building an OD matrix of implicit commuting flows, and compare it with a matrix from a travel survey.

Meaningful place detection works at the individual level and, in our method, requires manual input to define the method parameters. A higher order concept is land use, which identifies a place according to how individuals perform activities on it. Land use is a crucial factor in the design of transport systems and infrastructure; thus, it is important to measure and monitor. In our context, the availability of mobile traces enable the estimation of *land use profiles* based on how many mobile devices are connected to each BTS [17,21,22,23,24,25]. This can be done by estimating time-series of floating population per area of interest, and then clustering or decomposing those time-series using different methods: Eigen-decomposition [24], *k*-means [25], DBSCAN [21], network approaches [22], and classification using *Random Forests* [23]. External datasets (e.g., zoning codes, business location information, or social networks) can be used to either train the models [23], as well as explaining or classifying the clusters [21]. These methods have proven to be consistent across different cities, allowing urban planners to compare cities according to their land use patterns [22], as well as to study how rhythms of life differ according to socio-cultural factors [17]. Usually these *land use profiles* are built using weekly information (except in the case of [17], where daily rhythms were also estimated). In this paper we work with daily profiles, which are more narrow in terms of time than longitudinal studies (e.g., months [5] and even years [26]). However, daily profiles are arguably equally rich in terms of discovering land use patterns due to the richness of CDR data. We build daily profiles and perform *Agglomerative Clustering* [27], which allows us to study land use according to hierarchical categories instead of a fixed number of clusters or communities. By using this clustering method we do not need to assume properties of the floating population—the time-series by themselves provide enough semantic information. A hard problem for clustering techniques is to explain or label the obtained clusters. In our context, this has been done manually by using local expert knowledge [22,25]. We propose to use an external knowledge base of points of interest, and estimate information association metrics for them and each cluster. Particularly, we estimate *Pointwise Mutual Information* (PMI [28]), in a similar way as clusters of text documents have been labeled [29]. By combining hierarchical clustering and labeling, we can provide rich input to discovery of functional areas in the city [30,31].

As external knowledge base we use *OpenStreetMap* (OSM). Even though in previous work FourSquare data has been used in this context [21], this social network has been found to be very biased [32]. OSM, while not perfect in that aspect [33], has been found to have good coverage when contrasted with ordnance surveys [34]. Its availability in different parts of the world makes it a good dataset to label city-level spatial clustering.

## 3. Methods

In this section we explain how to aggregate and analyze citizen movement according to their connections to mobile antennas. We seek to solve the following problems:

1. Given a set of antennas A and a network event *e* for a device *m* in a CDR dataset D, estimate a geographical position $p_{m}$ based on the corresponding antenna *a* (Section 3.1).
2. Given a designed zoning Z, and the set of geographical positions P estimated during a day for all mobile devices, estimate the home and work zones $z_{h}$ and $z_{w}$ for a mobile device in (Section 3.2).
3. Given a designed zoning Z, and the CDR dataset D, estimate the set of land-usage clusters C, where each cluster *c* contains a set of zones $Z_{c}$. Then, characterize each $Z_{c}$ according to the Points of Interest (POIs) located in those zones (Section 3.3).

The first problem is motivated by the limits of geolocation in previous work. The second and the third problem are common tasks performed by urban planners, and we propose to evaluate them considering how results differ when using AVP and when not.

### 3.1. Antenna Virtual Placement

The mapping of network events to geographical positions can be done at several resolutions. Using a tower or BTS resolution, all devices are mapped to the geographical positions of the underlying BTS where their events are registered [3,35,36]. Although simple and straightforward, this approach does not reflect the true position of each device, and also uses a comparatively small number of sample points in the map where events are being placed. Our proposed approach, denoted *Antenna Virtual Placement* (AVP), consists in decoupling the antennas from their BTS by projecting them to the ground using specific geometrical parameters. In this way, the number of sample points in the map can be increased proportionally to the number of antennas or sectors associated to each base station, reducing the positioning error (distance between the antenna and the device). This approach follows ideas from [37,38], where the decoupling stage is performed only using the azimuth of each antenna (degrees with respect to the north direction).

AVP initially performs a linear projection of each antenna to the ground, using the down-tilt (degrees of inclination with respect to the vertical tower) and azimuth of the antenna, and the height of the tower. Then, using a Voronoi tessellation centered in the projected antennas, it optionally relocates the positions to the centroids of the tessellation polygons.

**Figure 1:**
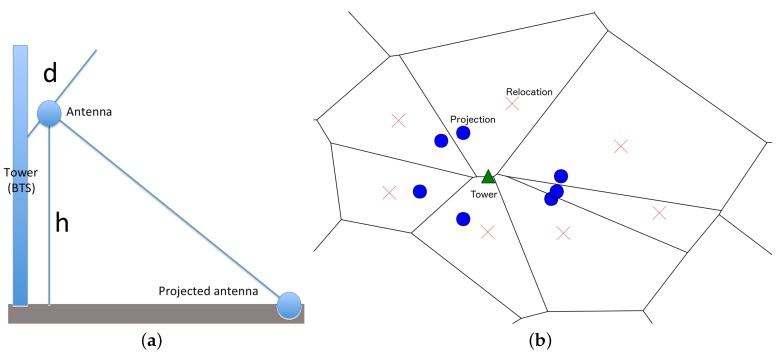
(**a**) Downtilt (*d*) and height (*h*) of an antenna placed in a tower or Base Transceiver Station (BTS) (side-view); (**b**) Sample of the Antenna Virtual Placement (AVP) decoupling process using a Voronoi tessellation to relocate the projections (top-view). The tower (green triangle) projects each antenna to the ground (blue circles), and each projection can be relocated to the centroid of Voronoi polygons (red crosses).

The steps of the method are as follows:

1. Define the set of antennas *A* and towers T, where each tower *τ* consists in a subset of antennas and a single antenna belongs to a unique tower. All antennas belonging to the same tower *τ* have the same geographical position of the tower, denoted $p_{\tau }$. Using the same notation, we have $p_{a}=p_{\tau }$ for all a∈τ.
2. For each antenna a∈A, obtain the azimuth *α*, downtilt *d* and height *h* (see Figure 1a).
3. Obtain the projection of the antenna *a*, denoted π(a), in the ground using the parameters (α,d,h) with simple trigonometric rules. The projection will have the new position $p_{\pi (a)}$.
4. Optionally, a relocation of the new positions can be obtained by moving the position $p_{\pi (a)}$ to the centroid of the Voronoi polygon generated with the positions $\{p_{\pi (a)}$: a∈A}. The relocated position is denoted $r(p_{\pi (a)})$ Each position $p_{\pi (a)}$ will belong to a unique Voronoi polygon, so the relocation can be viewed as a bijective map.
5. For each network event *e*, where the active antenna is *a*, set the position of the mobile device *m* as $p_{m}=r\left(p_{\pi (a)}\right)$.

In Figure 1b, we can observe a BTS and its corresponding antenna projections in the ground, and the optional relocation. We propose that this approach reduces the intrinsic error associated to the estimated position, in terms of the true position of a device.

### 3.2. Important Places at Individual Level

The study of transportation patterns tend to share general principles when estimating important places for OD matrices, particularly when considering *origins* as *home locations*, and *destinies* as *work locations* (note that work locations include educational and commercial activities). For instance, they use historical data to generate a classification model, and then try to find patterns in repetitive behavior in monthly time windows [10,39,40]. In this context, a regular trip is defined if the network events show regularity in their frequency [40]. We propose to consider only the regularity within a single day. This would allow to compare different days and obtain highly granular analysis of urban patterns.

To exploit the structure of regularity in movement, we assume that the majority of citizens work during the day, and spend most of the time at the physical locations of their workplaces. This can be reflected on how network events accumulate at nearby antennas. In our case, we use the CDR data from a single day, and fit a probability distribution to the frequency of connections to each antenna during specific time windows. To define if the subscriber of a mobile device is at an important place, we proceed as follows:

1. Define time windows during the day that are likely to be related to home/work. For instance, to model home location we consider two time windows: one in the range 6:00 A.M. to 8:00 A.M., and one during 8:00 P.M. to 10:00 P.M.
2. In all defined time windows, we weight network events using the exponential distribution:
  $$f\left(x;\gamma \right)=\gamma e^{-\gamma x}$$
  where *x* is the position of the network event in the time window, and the value of the parameter *γ* is determined according to the threshold given as confidence interval. If we consider a 95% interval, then f(N;γ)=0.05, having *N* as the number of records under consideration. Thus, the sum of weights for all records in a given time window is always 1.
3. For each mobile antenna *a*, we estimate $P(a_{t})$ as the sum of the weights of its corresponding records in a specific time window *t*.
4. To determine the regularity of citizen behavior in the different time windows, we use an intersection metric converted to a distance. To calculate this distance, we define *a* as an antenna and $P(a_{t})$ as its weight in the time window *t*:
  $$d\left(t_{1},t_{2}\right)=1-\sum_{a\in A}\min\left(P\left(a_{t_{1}}\right),P\left(a_{t_{2}}\right)\right)$$
  where *A* is the set of antennas. Note that *d* is 1 when there is no overlap or similarity between distributions, and 0 when all distributions are equal. Empirically, we have found that a d≤0.4 is a good threshold for regularity.
5. The device home/work locations are the weighted interpolations of the antenna positions ($p_{m}=r\left(p_{\pi (a)}\right)$, estimated with AVP at Section 3.1) the mobile device was connected to in the corresponding time windows.

Having these predicted important places, it is possible to build an aggregated OD matrix where we consider source and target areas, which can be administrative locations as well as designed zones. In this paper we work with the latter, while in prior work we worked with the former [41].

### 3.3. Determining Land-Usage Patterns

In the previous section we worked with important places at an individual level, or, as designated on previous work, *anchor points*. For instance, [17] defined three kinds of anchor points: *home*, *work*, and *free time*. At a higher level, city areas that tend to concentrate similar kinds of places can be classified according to their land use. There are at least two kinds of categories: *dormitory* and *non-dormitory*, where dormitory locations are residential areas whose primary use is to provide a home location for the working population, but that do not have industrial nor educational activity during a day. Non-dormitory, however, is a complex category: Is it commercial only? Is it mainly about business districts? There is a rich variety of possible uses of non-dormitory areas [22]. We test if by using clustering methods we can find those areas.

Having diversity of temporal typology in mind, our method builds clusters of zones of the city based on the level of activity in each location, having as input a smoothed count of mobile phones connected to the antennas in those locations. From [22,25] we borrow the notion of activity profile for those smoothed counts. Because we use agglomerative clustering we do not need to select a specific number of clusters; instead, we can flatten our hierarchy of clusters as needed.

Our proposed method can be formalized as follows:

1. We analyze network events at zone level. For each zone *z* (which may or not may be designed), we build a time-series $Y_{z}$:
  $$Y_{z}=\left\{C_{t,z}:t\in M\right\}$$
  where *M* is the set of minutes of the day, starting from 00:00 and ending at 23:59, and $C_{t,z}$ is the number of mobile phones that generated network events at time *t* in the set of antennas $A_{z}$ assigned to zone *z*, using AVP (Section 3.1).
2. For each time-series $Y_{z}$, we build a smoothed time-series $S_{z}$ using LOWESS (*Locally Weighted Scatterplot Smoothing*) interpolation. This allows us to smooth noise and drastic changes in the number of connections in consecutive intervals of time, as well as to interpolate the number of network events between minutes. This is needed because CDR data is sparse.
3. To quantify how near (or similar) the time-series are we build a pairwise distance matrix *M*. Each element $M_{u,v}$ contains the *correlation distance* (as in [25]) between time-series *u* and *v*, defined as follows:
  $$M_{u,v}=1-\frac{\left(u-\bar{u}\right)\cdot \left(v-\bar{v}\right)}{{\left||(u-\bar{u})|\right|}_{2}{\left||(v-\bar{v})|\right|}_{2}}$$
  where $\vec{u}$ is the mean of the value of time-series *u*, and x·y is the dot product between time-series *x* and *y*.
4. Having the pairwise distance matrix between all smoothed zone time-series, we estimate agglomerative clustering using Ward variance minimization [27]. As result, we have a dendrogram of locations.
5. We flatten the dendrogram of locations. If the number of desired clusters is known, the flattening can be performed based on *cophenetic distance* [42] between locations. This distance is the height of the dendrogram where the corresponding location branches merge into a single branch.

Usage of the activity profiles allows us to create a temporal typology of city areas. The next step is to explain the obtained clusters using as input a list of Points of Interests (POIs) or venues. A POI is a specific point in the map that encodes the geographical position of a place that is visited by people to perform activities (e.g., a business, a landmark, a metro station, and so forth). Thus, each POI belongs to specific categories that can be used to explain clusters, by estimating which categories are associated to each cluster. As metric of association between venues and zones we use *Pointwise Mutual Information* [28]. PMI measures the relationship between the joint appearance of two outcomes (*x* and *y*) and their independent appearances. It is defined as:

$$\mathrm{PMI}\left(x,y\right)=\log\frac{P(x,y)}{P(x)P(y)}=\log\frac{P(x|y)}{P(y)}=\log\frac{P(y|x)}{P(x)}$$

where, in our case, *x* is a POI category (e.g., *company*) and *y* is a cluster of zones, as defined earlier. Note that PMI is zero if *x* is independent of *y*, it is greater than 0 if *x* is positively associated with *y*, and it is lesser than 0 if *x* is negatively associated with *y*. We characterize a cluster *y* by analyzing the set of associated POIs $X_{y}$, defined as:

$$X_{y}=\left\{x:P\left(x|y\right)>0\right\}$$

Having $X_{y}$ for each cluster, we qualitatively elaborate an explanation of it, based on both features: its characterization and the shape of its time-series.

## 4. Materials

In this section we describe the materials used to apply and evaluate our proposed methods. Particularly, we use a travel survey published by the Secretary of Transport Planning (http://www.sectra.gob.cl/) in Chile, and a set of mobile antennas and a CDR dataset from Telefónica (http://www.movistar.cl/), the largest telecommunications company in Chile, with a market penetration of 38%. Santiago, the capital, is a city with almost 8 million inhabitants, with a surface of 867.75 square kilometers, and with an integrated public transport system named Transantiago. The Metropolitan Area of Santiago that we study in this paper is composed of 35 independent administrative units denoted municipalities.

### 4.1. Santiago 2012 Travel Survey

The Santiago 2012 travel survey (ODS hereafter) was performed during 2012–2013 [43]. It contains 96,013 trips from 40,889 users. This ODS, used to define public policy related to transportation in the city, is performed every 10 years due to its costs.

The information of trips is obtained through the travel diaries fulfilled by the surveyed persons. The diaries include other municipalities outside the area, as well as cities in other regions, due to the characteristics of the sampling method. Additionally, the survey defines a designed zoning of the city, with 752 zones within the considered municipalities. Each zone intends to control for land use and population density. The mean number of zones per municipality is 21.03 (σ= 10.58, min= 1, max = 52, median = 18). A zone belongs only to one municipality. Even though each trip in the survey is associated to a zone and a municipality, the survey is only representative at municipal level. At its current granularity, the data available is not enough to calculate reliable mobility patterns at zone level.

**Figure 2:**
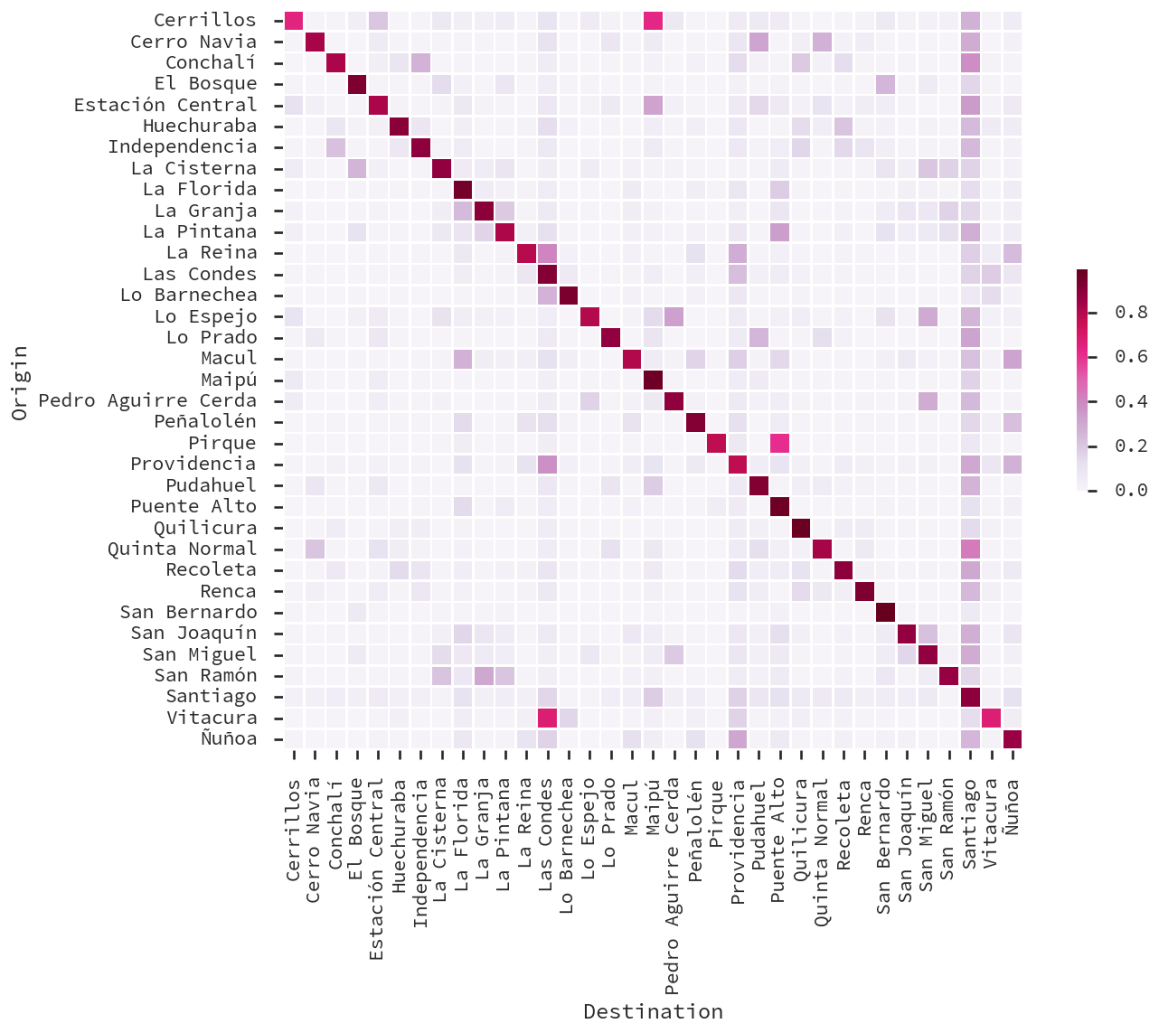
Origin-Destiny (OD) matrix of municipalities for Santiago, according to the Origin-Destiny Survey 2012.

In this paper we focus on the 51,819 trips performed on working days, from 22,541 surveyed inhabitants of the 35 municipalities under consideration. We aggregated those trips according to municipality into an OD matrix, shown in Figure 2. One can see that there are municipalities that tend to receive more trips than others. This is because most of the commercial and working land use is on the municipalities of *Santiago*, *Providencia*, *Las Condes* and *Vitacura*. Note that the municipality of Santiago is at the center of the Santiago Metropolitan Area.

### 4.2. Mobile Antennas

**Figure 3:**
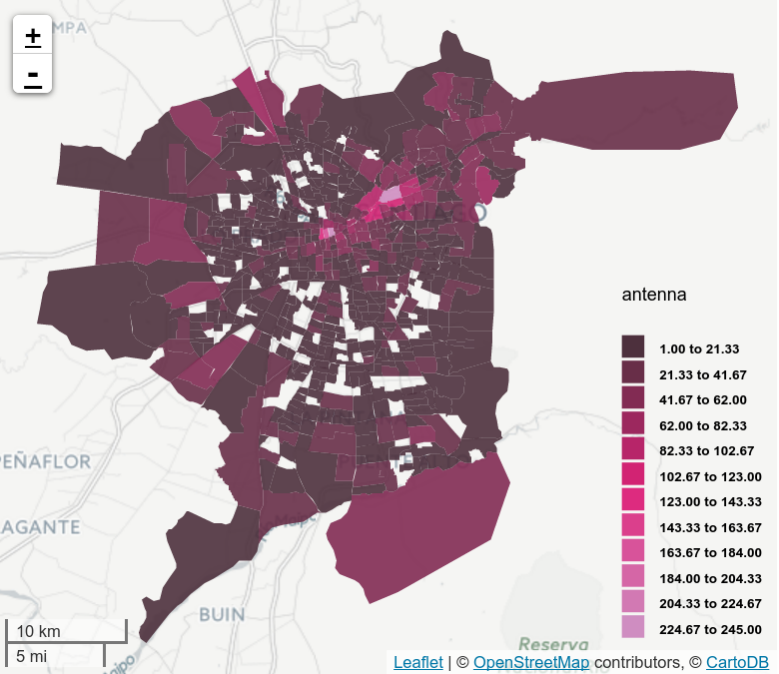
Antenna coverage on the 752 zones from the ODS zoning, using the corresponding BTS positions. Colors indicate antenna density in each zone.

In the 35 municipalities under consideration, Telefónica has 13,860 antennas (from 1464 towers) with different mobile technologies: 2G (GSM), 3G (UMTS) and 4G (LTE). Figure 3 displays the antenna territorial density, without decoupling the antennas from their corresponding tower. Note that the antenna distribution is not homogeneous on the city, nor at municipal level. For instance, antenna distribution is rank-correlated with the ODS, considering aggregated origins and destinations at municipal level (ρ=0.91, p<0.001, in both cases).

### 4.3. Call Detail Records

As mentioned in the previous section, the methods in this paper are proposed with the intention of performing daily-based analysis. We consider mobile traces extracted from anonymized CDR network events: calls, text messages, and data events for all Mondays and Tuesdays of June 2015. In the Appendix we have included a discussion about the suitability of data events for this particular dataset. A daily-characterization of this dataset was performed in [44], where it was found that different days exhibited similar mobility patterns, except in two particular days where unexpected (e.g., a public transport strike) or uncommon things happened (e.g., a soccer match from a latin-american tournament). Moreover, the entropy and frequency of events in each day presented equal distributions in all days except the previous two.

The definition of entropy used in the analysis is the *Shannon entropy* of a mobile device *u*:

$$H_{u}=-\sum p_{i,u}\ln p_{i,u}$$

where $p_{i,u}$ is the probability that user *u* has a network event in the *i*th hour of the day. The purpose of estimating entropy is to have a measure of diversity with respect to time for each user.

**Figure 4:**
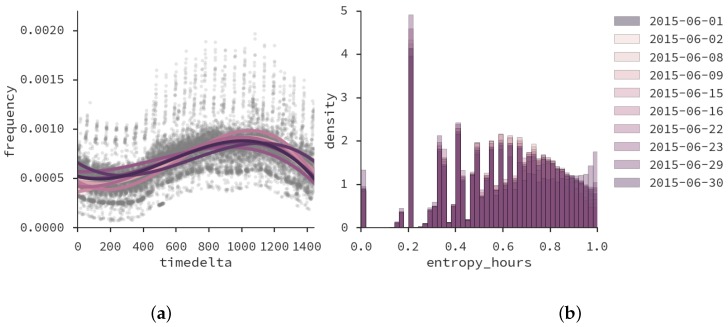
Distributions of Call Detail Records (CDR) event frequency (**a**), and entropy with respect to hours of the day (**b**). Source: [44] (used with permission).

In terms of frequency and entropy, Figure 4 displays their distributions for all days in the dataset. In Figure 4a each dot is a minute in a specific day, and the frequency encodes the fraction of events that the dot contains per day. One can see that the distribution of frequency of events can be approximated by a cubic curve, with a higher frequency of events in the afternoon. This higher frequency is expected, because network events from Internet connections are more common in the afternoon, due to the activities performed on the city. This regularity in daily behavior implies that a daily-based analysis is feasible.

Figure 4b displays the distribution of user entropy with respect to hours of the day, for each day. This chart also exposes the regularity in daily behavior, except for a couple of days that had unexpected events (one had a strike of transport workers, and another had a massive sports event). It is interesting to note that those events did not change event frequency—just the distribution of entropy.

In this dataset we use the same entropy filtering as in [44]: we consider only those devices with entropy between the 0.4 and 0.9 quantiles of the distribution, as a way to analyze inputs with enough records to be considered active, but not enough to be suspicious of not being a human-held device. Note that we do not disclose the exact number of users in each day due to confidentiality and commercial issues.

### 4.4. OpenStreetMap

To characterize land use we resort to crowdsourced geospatial information available on the OpenStreetMap platform [45]. We downloaded a database dump from November 2015 that contains all venues and POIs with geographical coordinates belonging to Chilean territory, as well as road network information (this database is available at http://download.gisgraphy.com/openstreetmap/pbf/). We only consider POIs and venues in this paper. From now on, we refer to POIs and venues indistinctly.

In total, the dataset contains 292,239 POIs. In addition to considering only those venues that are inside the designed zonification, we also took into account only the following types of venue: *amenity*, *building*, *craft*, *emergency*, *historic*, *landuse*, *leisure*, *man_made*, *office*, *power*, *shop*, *sport*, *tourism*, *public_transport*, *place*, *barrier*, *highway*, *military*, *natural*, *railway*, *route*, *waterway*, and *landmark*. Each of these venues may have a special tag to specify the kind of venue.

**Figure 5:**
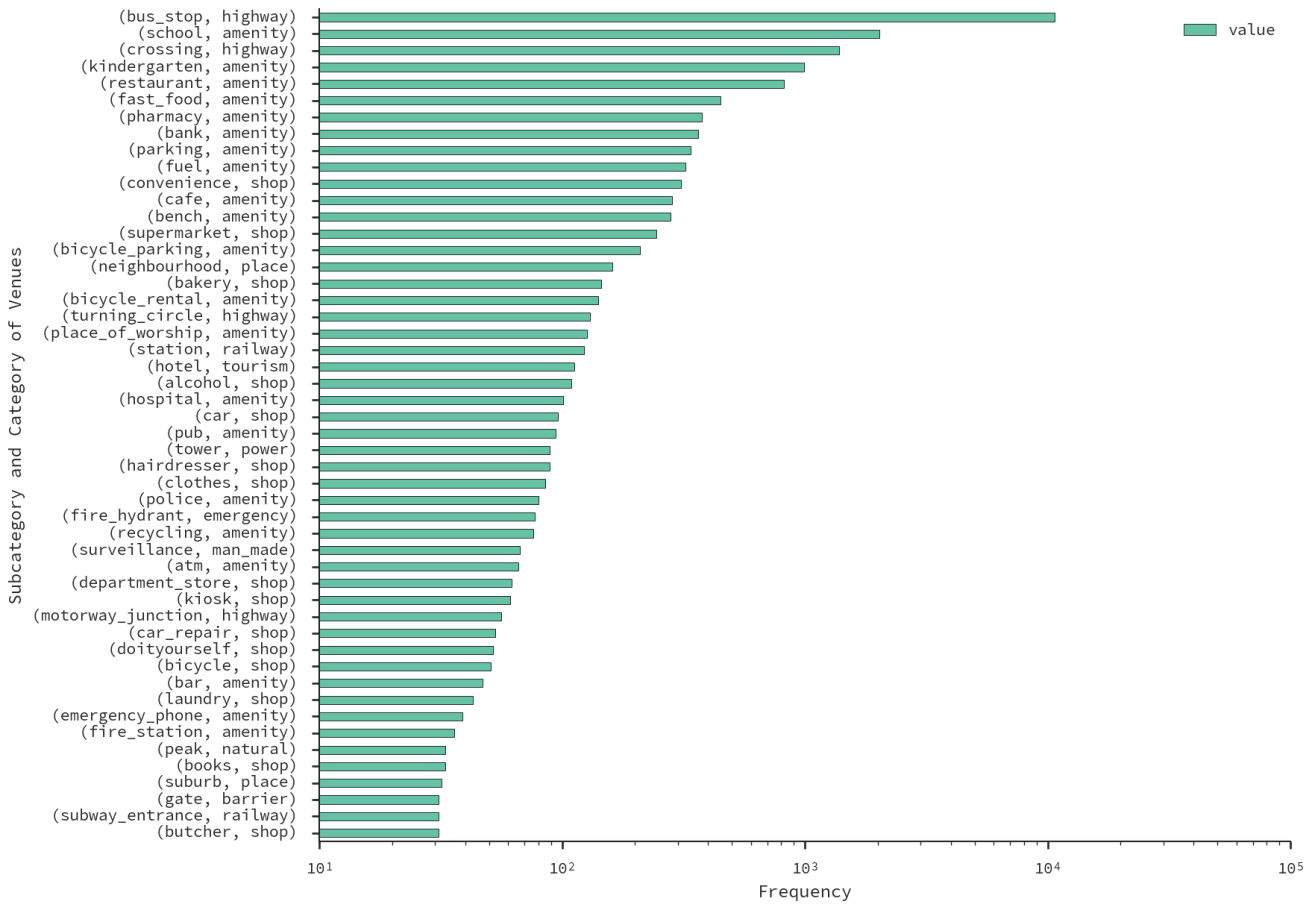
Number of *OpenStreetMap* (OSM) venues per studied sub-category in the Santiago. The labels include secondary category and primary category of each venue type.

The dataset contains 22,980 venues that are located within the studied zones. The top-three are *highway* (12,299), *amenity* (7,689) and *shop* (1761). Note that to have a deeper level of information, we consider venue subcategories in further analysis, due to them being arguably more informative. Figure 5 displays a histogram of the top-50 subcategories. The most common venue is *bus_stop*, with a first-level category of *highway*. This shows the importance of public transport in the city.

**Figure 6:**
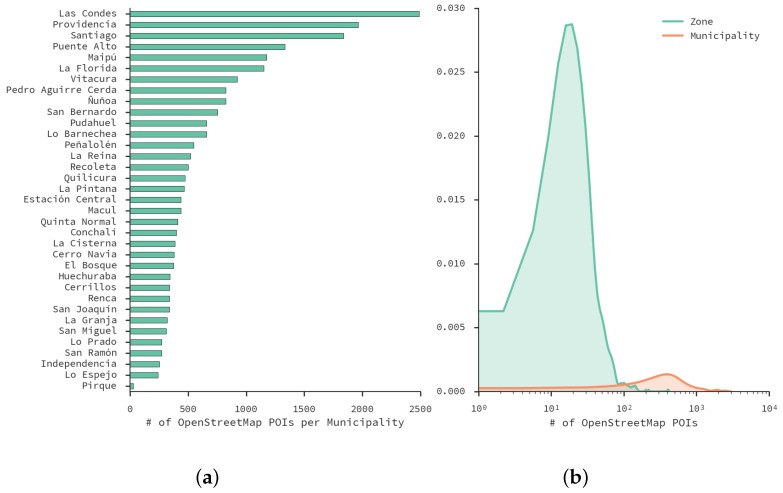
(**a**) number of *OpenStreetMap* (OSM) venues per municipality in Santiago; (**b**): distributions of the number of venues per designed zone and municipality.

Figure 6 shows the distribution of venues in municipalities. Figure 6a is a histogram that displays which municipalities has more venues—*Las Condes*, *Providencia* and *Santiago* are the top-three. This is expected because those municipalities are within the most common destinies according to the ODS. Figure 6b shows the distributions of venues per zone and municipality. One can see that the zone distribution is very skewed. In fact, the mean number of venues per zone is 30, while the maximum is 412. Given that OSM venues cover all municipalities and 99.7% of zones (only two do not have venues in them), it is feasible to use OSM data to explain land use patterns found using our method.

## 5. Case Study: Santiago, Chile

In this section we report the results of applicating our proposed methods into our CDR dataset.

### 5.1. Antenna Virtual Placement

**Figure 7:**
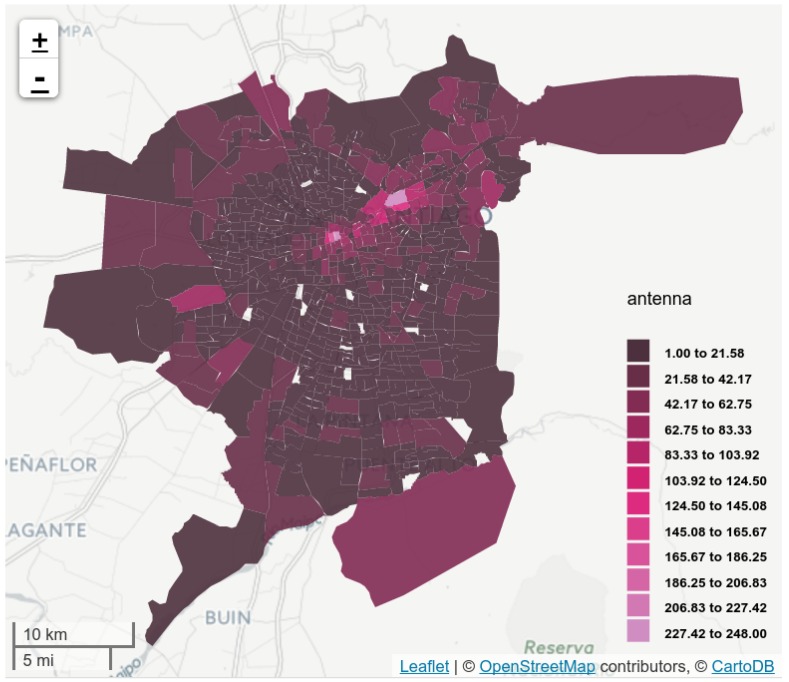
The 752 zones from the OD Survey zoning. Colors indicate antenna density in each zone according to geographical positions obtained using the AVP method.

AVP diversified the assigned positions to each antenna, improving coverage of the city at the zone level. This can be explained by the map in Figure 7, where it can be seen how the zone coverage has improved in comparison to Figure 3. For instance, the number of covered zones increased from 636 to 736 (of 752). The medians and means of antennas per zone have also decreased (from 15 to 12, and from 20.11 to 17.58, respectively).

**Figure 8:**
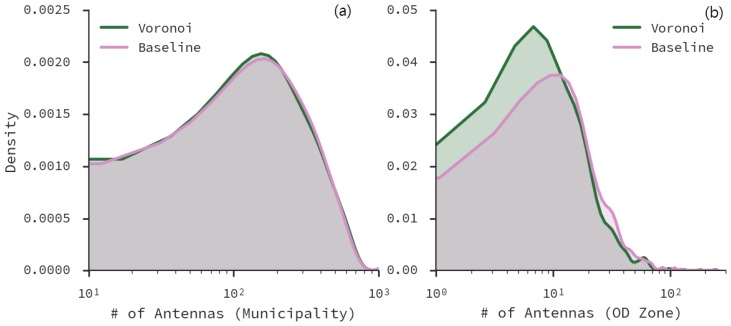
(**a**) Distribution of antenna positions per municipality; (**b**) Distribution of antenna positions per OD zone.

We estimated the distribution of the number of antennas per area of interest using Kernel Density Estimation (KDE). Figure 8 shows the KDE distributions of antenna coverage per municipality (Figure 8a) and OD zone (Figure 8b) after applying AVP to decouple the antennas located in the city. The distributions are identical at the municipality level, but the distribution at zone level shows a different shape, confirming the decrease observed on the means and medians of number of antennas per zone.

**Figure 9:**
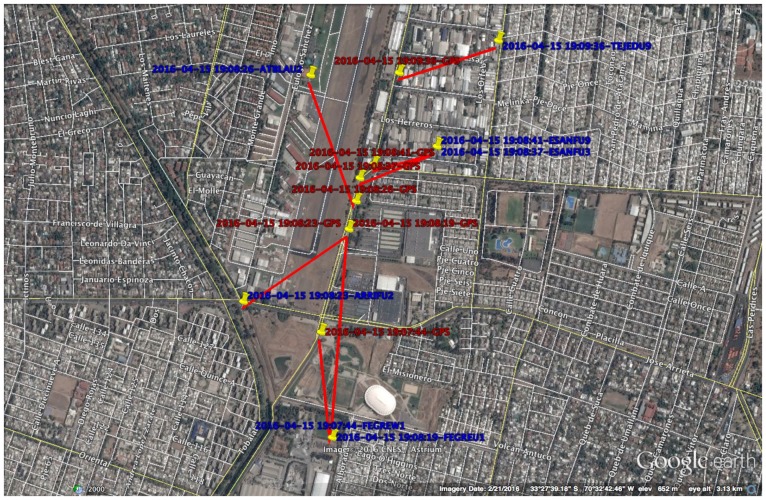
GPS tracking to measure the difference between the device location (red labels) and the antenna location (blue labels). In this image the antennas are located in the position of the corresponding BTS.

**Figure 10:**
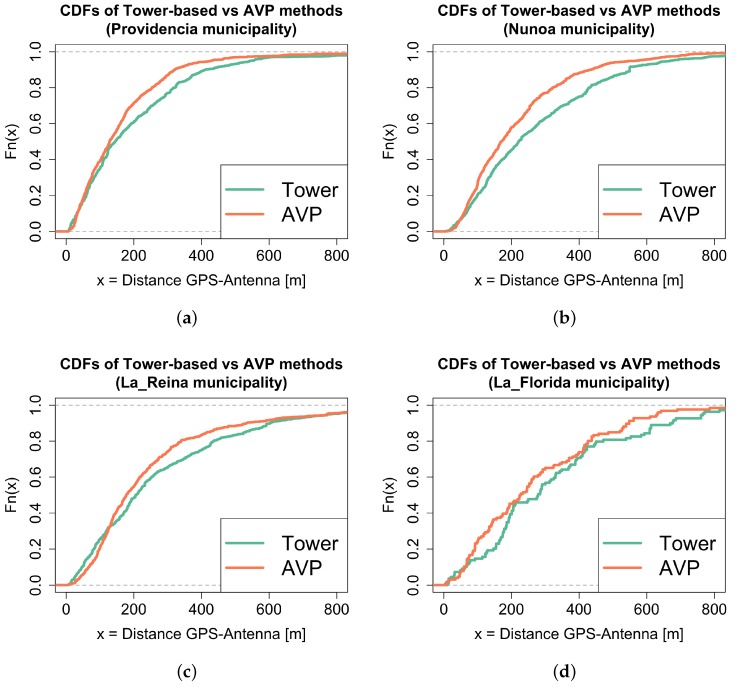
Accuracy measurement of the AVP and BTS-based positions with respect to ground-truth GPS positions of mobile devices. Particularly, we compare the cummulative distributions of distance between those methods and the ground-truth. The top row contains two municipalities with high antenna density: *Providencia* (**a**) and *Ñuñoa* (**b**); the bottom row contains two municipalities with low antenna density: *La Reina* (**c**) and *La Florida* (**d**). In all cases, AVP reduces the error introduced by using BTS-based positions to approximate device location.

To measure the accuracy of the device position using AVP, we collected a set of field GPS measurements in several municipalities of Santiago, with different antenna densities (Figure 9). Only GPS measurements with less than 50 m of intrinsic accuracy were included. In Figure 10 we observe the cumulative distribution functions (CDF) of the distance between GPS positions and antenna positions for each measurement. Each CDF is calculated using two antenna positioning methods, the simple BTS-based method and AVP. After applying the Kolmogorov-Smirnov test to each pair of distributions, we consistently rejected the null hypothesis which claims that the GPS-Antenna distances using the tower-based method were stochastically smaller than the distances obtained using the AVP method (p<0.001 for the municipalities of *Providencia*, *Ñuñoa* and *La Reina*, and p<0.05 for *La Florida*; the sample number of distances GPS-Antenna in each case is 360, 287, 188 and 83 respectively).

### 5.2. Important Places

**Figure 11:**
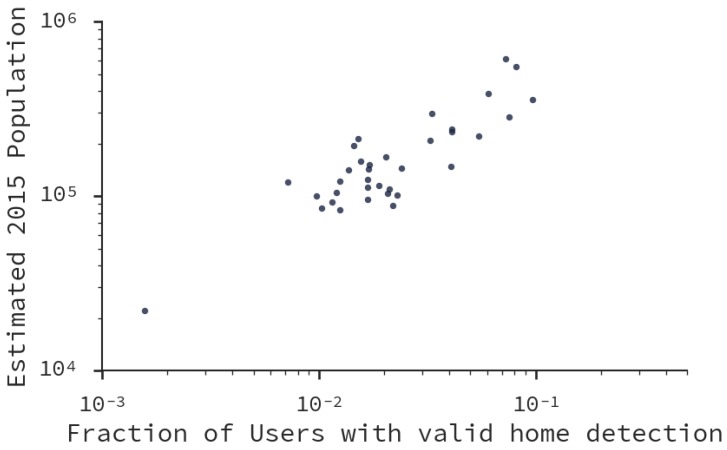
Scatterplot of the distribution of detected *home locations* and the 2015 estimated population for municipalities in Santiago. Both axis use a log-scale to account for the inequalities in population distribution. The non-scaled values show a Pearson correlation coefficient of r=0.84 (p<0.001).

We estimated home and work locations in the dataset. Particularly, without losing generality (due to the similar structure between days shown in Figure 4), we analyze the matrix obtained from 1 June 2015. Due to our reliability threshold, we obtained home and work locations for only 10% of the mobile devices. To evaluate the accuracy of this estimation we estimated the Pearson correlation coefficient *r* between the population projected for the year 2015 by the National Institute of Statistics in Chile [46] and the fraction of individuals per municipality. As result, we obtained r=0.84 (p<0.001), a very high correlation (see Figure 11). This means that our method captured the distribution of city inhabitants according to their municipalities, in spite of using just the 10% of the dataset.

**Figure 12:**
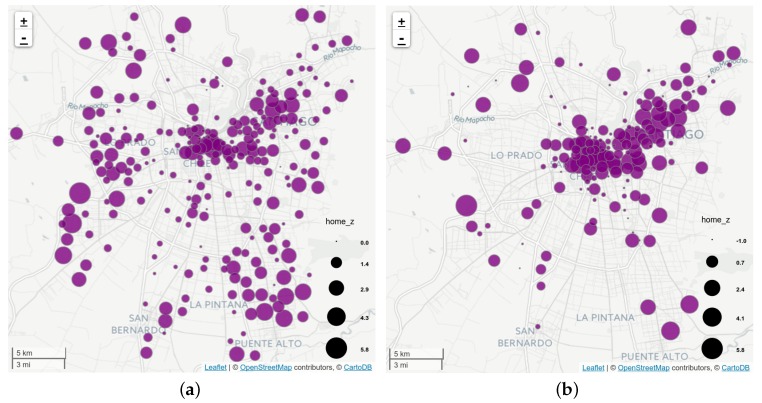
Zones from the city more likely to contain home (**a**) and work locations (**b**). The size of each bubble is proportional to the *standard score* of the population fraction detected to be within each zone for each category.

**Figure 13:**
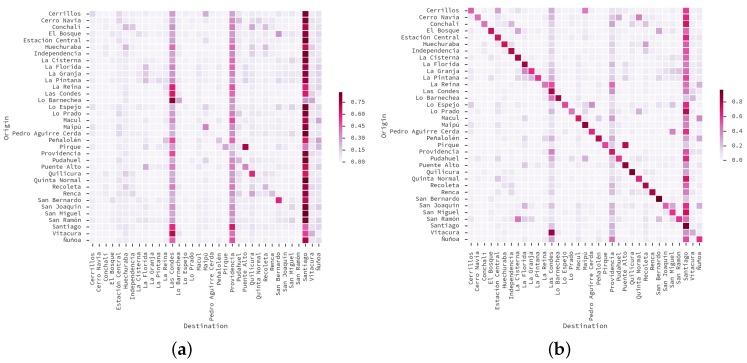
Comparison of OD matrices at municipal level, considering trips for work, study and shopping activities in workable days. (**a**) Matrix obtained using our method, with AVP mapping of positions; (**b**) Matrix with trips extracted from the ODS.

In Figure 12 we group users according to their assigned home (Figure 12a) and work zones (Figure 12b) from the designed zoning. Each zone contains a bubble encodes the *standard score* of the population fraction within that zone. Thus, Figure 12a shows the city zones that are more likely to be of residential use, while Figure 12b shows the city zones that are more likely to be work places. One can see that work locations are concentrated on the center and mid western areas of the city. Because a displacement from home to work locations imply a trip between them, we aggregated the trips in an OD matrix of municipalities, displayed on Figure 13a. Figure 13b shows the matrix from the ODS, considering only trips in working days with one of the following purposes: *to work*, *to study*, and *shopping*, as those are the kind of trips that can be captured by our proposed method.

In our matrix, the top-5 originating locations are *Santiago* (9.68% of the trips), *Maipú* (8.09%), *Las Condes* (7.51%), *Puente Alto* (7.29%) and *La Florida* (6.03%). The top-5 destinations are *Santiago* (24.69% of the trips), *Las Condes* (14.28%), *Providencia* (14%), *Ñuñoa* (4.06%) and *Vitacura* (3.47%). To evaluate how similar is our matrix to the OD Survey 2012, we estimated the Spearman rank-correlation of all source-target pairs of both matrices, obtaining ρ=0.81 (p<0.001). This is an improvement over our previous work, where we did not perform AVP, and our correlation was ρ=0.70 (p<0.001) [41].

### 5.3. Land-Use Results

**Figure 14:**
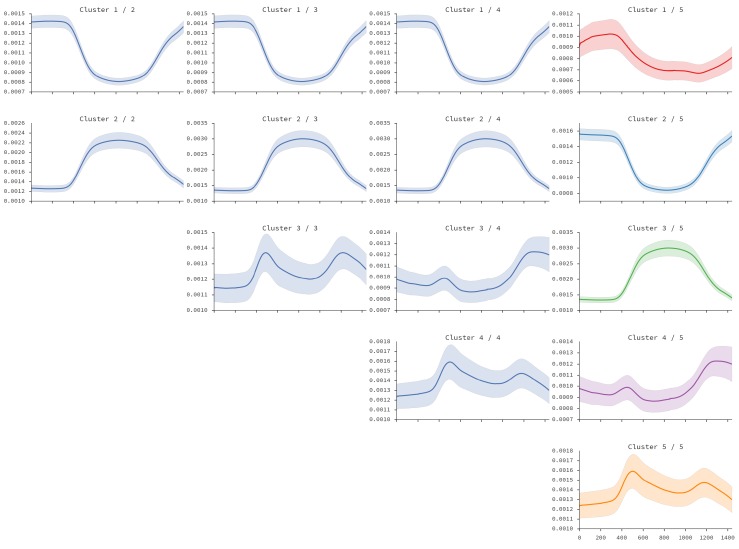
Clusters obtained from the analysis of district dynamic population.

We performed agglomerative clustering over the smoothed distributions of floating population for each designed zone. Figure 14 shows the obtained clusters after flattening from two to five clusters. One can see that at two levels (the first column) there are two clear clusters: dormitory and non-dormitory. The dormitory cluster is characterized with a high floating population at sleeping hours, and a very low floating population at working hours. As expected, the non-dormitory cluster shows the opposite behavior. In the second column, representing three clusters, one can see that the new cluster added is extracted from the initial non-dormitory cluster, representing locations with higher traffic just before and after working hours. These shapes are very similar to the daily rhytms detected in [17]—there, the third curve is characterized as “movement”, indicating that it contains activities performed during the transition from home to work (and viceversa). At the next level, in the third column, this cluster is separated into two: those with more activity after working hours (third row), and those with more activity before working hours (fourth row). Finally, in the last level with five clusters, the dormitory cluster is split into two, revealing that there is a cluster of locations that show an increased floating population just before working hours. In the remainder of this section, we will consider these five clusters for analysis. We estimated a higher number of clusters but the differences between them were becoming too small for our analysis. However, note that previous work [22,25] worked with four clusters, although they worked with weekly data, while we work with daily data and still obtained a number of comparable clusters.

**Figure 15:**
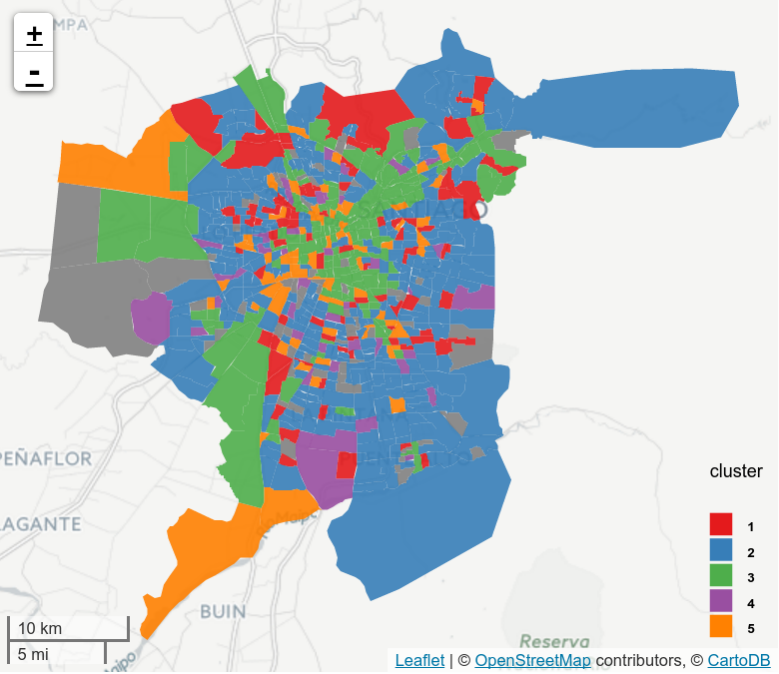
Clusters obtained from the analysis of floating population based on mobile connectivity.

**Figure 16:**
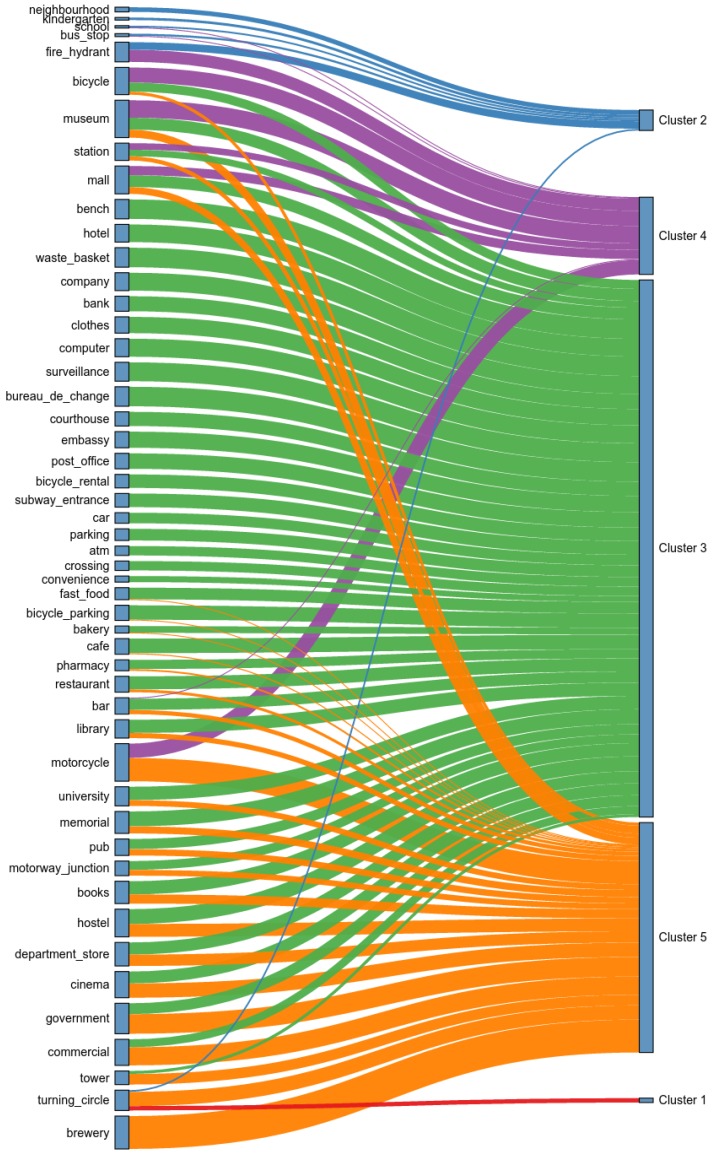
Flow diagram of OSM Points of Interest (POI) sub-categories and their association (through Pointwise Mutual Information (PMI)) with each cluster.

Figure 15 shows the spatial distribution of the clusters. One can see that zones belonging to the same clusters tend to appear together, implying a geographical pattern driven by floating population flow. To characterize land use, we estimated PMI for all venue subcategories in OSM and clusters in the dataset. However, we use only the top-50 venue subcategories in terms of informativeness. Those venues were selected using univariate feature selection with a chi-square test. The chi-square test measures dependence between stochastic variables, which, as a consequence, removes the venues that are the most likely to be independent of cluster and therefore irrelevant for classification. Since the feature selection keeps only the top-50 scores, we also maintain features that have enough frequency to be interesting for analysis (e.g., an airport, while highly distinctive of one cluster, is not frequent and thus it does not help to discriminate between other clusters). Figure 16 shows the selected 50 features and their association with each cluster using a flow diagram. The PMI scores of the most associated venues to each cluster are shown on Table 1.

Table 1 also shows the fraction of the city surface corresponding to each cluster, as well as a label assigned by us based on the most associated venues (or lack thereof). The biggest cluster is #2, labeled *Dormitory*. It is a residential cluster, associated with neighborhoods. This is coherent with its activity profile on Figure 14. The second cluster is #3, labeled *Business*. It is a cluster of low population at early morning and night, but during the day it has a considerable floating population. It has commercial venues, but the most associated POI is *waste_basket*, possibly due to the fact that many people are transiting from one place to another within the same cluster because of commercial, work and study activities, as well as commuting. The third cluster is #1, labeled *Transition*. Its activity profile distribution shows a similar profile to dormitory areas, but with increasing population in early morning, and a deep decrease in population at working hours. According to its associated venues, only *turning_circle* has a possitive association. Thus, we define these areas as transition areas between dormitory and non-dormitory locations. Clusters #4 and #5 are smaller than the others. We labeled them as *Leisure Acitivities After Working Hours* and *Civic Districts and Recreation Activities Before Working Hours* due to their activity profiles and their associated venues. According to Figure 14, when using four clusters instead of five, both clusters merge into one. Their separation into before- and after-activities may be interesting for analysis in future work.

## 6. Discussion and Conclusions

Our results indicate that it is possible to work with CDR daily data and gain understanding of urban patterns. In this section we discuss further the implications, limitations, future work and concluding remarks of this paper.

### 6.1. Implications

Our contributions have two-fold implications. First, we proposed a way to improve coverage of mobile device geolocation based on antenna connectivity. This method, named Antenna Virtual Placement, improved the results of our methods, initially described in [41], and deepened in this work. This improvement is explained by the better surface coverage obtained using AVP. This implies that methods that work on CDR data and that do not use AVP could be improved just by including this pre-processing step into their pipelines. The AVP approach represents a good compromise between desired accuracy and technological complexity of the implementation.

Second, previous work has analyzed weekly and monthly data, while we work with daily data. We obtained a very high correlation of important places with a survey based OD-matrix, and our results are quantitative and qualitatively comparable to previous work in terms of land use analysis. This implies that daily data has potential to aid and increase the understanding of a city. Moreover, our proposed methods were simple, yet not trivial, and so they can be easily implemented. For instance, we have used agglomerative clustering, which allows us to merge and separate clusters according to analytical needs. Urban planners who want to work at city level might use few clusters to understand the greater, general patterns, and those who want to work at local (municipal or even zone) level can go further in the cluster hierarchy. Tasks that benefit from our results are recommendation of places and routes, retail store placement, evaluation of environmental impact from a land-use perspective, estimation of transport effects from pollution alerts, and so on.

The usage of OSM proved useful when characterizing clusters using associativity metrics. For instance, we found that the dormitory cluster is associated with *fire hydrants*, while the business cluster does not have that strong association. One of the reasons that explains this association is that residential areas have many houses and apartment buildings that, due to their height, do not require to have dry standpipes by law, unlike buildings in business districts, which are much more taller and are supposed to concentrate more people. Note that previous work has used FourSquare to perform similar analysis, but check-in based platforms do not contain that kind of POI information, as they tend to focus on popular places in business districts and recreational locations—it is unlikely that a fire hydrant is defined as a POI in social networks. Thus, we believe that our methods and results prove to be useful in the development of urban computing and ambient intelligence applications that exploit this crowdsourced information to, for instance, design urban policies to encourage land use change.

### 6.2. Limitations and Future Work

While AVP improves results, it still can be refined. For instance, instead of assigning a fixed position for all mobile devices connected to the same antenna, a probability distribution over the spatial signal coverage could be used. This would help to reduce even more the error in location assignment.

Another important limitation regarding AVP is that downtilt and height, two of input values, are not always available in open datasets. However this is a limitation on the availability of data and not of our proposed method. Both parameters can be approximated based on manual observation or topographic information.

In terms of important places, we assumed that displacements between those two places were trips, and we compared those trips with a ground-truth OD Survey. However, our method, even though it is capable of determining that a trip was performed, it cannot estimate travel time nor travel mode. To surpass this limitation, a trip detection method could be applied on the dataset instead, such as [44]. Moreover, we noted that the main differences between our matrix and the ODS can be seen on the matrix diagonals in Figure 13. The ODS contains many intra-municipality trips, which are short enough in distance for our method to capture.

Regarding clustering, we performed a very similar analysis to other works in the literature, and we did not add new features. While this approach has worked in the past, it does not find latent relationships between actual land use and the activity profiles. We explained/characterized clusters using volunteered geographical information—a different approach would have been to cluster based on both, in a similar way to taxonomy-based annotation [31]. However, this brings the problem of how to interpret the different kinds of features, and to compute a distance metric between designed zones based on the activity profile and the POIs present in it.

These limitations will be addressed in future work. Additionally, critics may rightly say that our methods to sensing the city are ad-hoc for Santiago. However, our results are coherent with those from previous work, as Santiago has a comparable to other cities previously analyzed (i.e., Madrid [21], Rome [15], Tallin [5], etc.). Moreover, work analyzing CDR data has focused either on developed, industrialized countries, or developing countries with poor transport infrastructure [47], as well as the comparison of both scenarios [48]. Being a growing city from a developing country [8], Santiago introduces an interesting mixture of availability of infrastructure and ground truth datasets. Our results complement previous work, while at the same time test alternative approaches to clustering floating population.

### 6.3. Concluding Remarks

In this paper, we presented methods to sense the city, starting from the Call Detail Records generated from mobile connectivity logs, to the determination of important places (work and home) for mobile users, and determination of land use, explained using crowdsourced data. Our methods provide improvements to previous results, as well as methods that can be included in other methodologies, particularly *Antenna Virtual Placement*. We discussed the implications of these results, as well as their limitations and future lines of work. Our conclusion is that sensing the city using Call Detail Records is possible even at the daily level, using non-trivial but simple and effective methods, even in big cities from non-developing countries. We provided methods for two common tasks from urban planning that can be used either as input or as part of urban computing applications.

## Figures and Tables

**Table 1:** Cluster information: assigned labels from their top-associated venues, as well as corresponding city surface.

Cluster ID	% of City Surface	Top-15 Venues	Label
Cluster 1	13.34	turning_circle (0.32)	Transition
Cluster 2	46.60	fire_hydrant (0.54), neighbourhood (0.34), kindergarten (0.19), bus_stop (0.15), school (0.13), turning_circle (0.12)	Dormitory
Cluster 3	24.66	waste_basket (1.40), bureau_de_change (1.40), bench (1.39), surveillance (1.35), computer (1.29), company (1.28), hotel (1.27), clothes (1.17), embassy (1.16), post_office (1.11), bank (1.08), memorial (1.07), hostel (1.06), cafe (1.04), bicycle_parking (1.03)	Business
Cluster 4	5.96	museum (1.25), bicycle (1.08), motorcycle (1.03), fire_hydrant (0.87), mall (0.66), station (0.47), bar (0.07), bus_stop (0.06), school (0.05)	Leisure Activities After Working Hours
Cluster 5	4.43	brewery (2.36), motorcycle (1.67), government (1.41), commercial (1.30), cinema (1.03), turning_circle (1.02), hostel (0.93), department_store (0.81), tower (0.76), books (0.66), museum (0.57), memorial (0.50), mall (0.49), pub (0.46), university (0.41)	Civic Districts and Recreation Activities Before Working Hours

**Figure A1:**
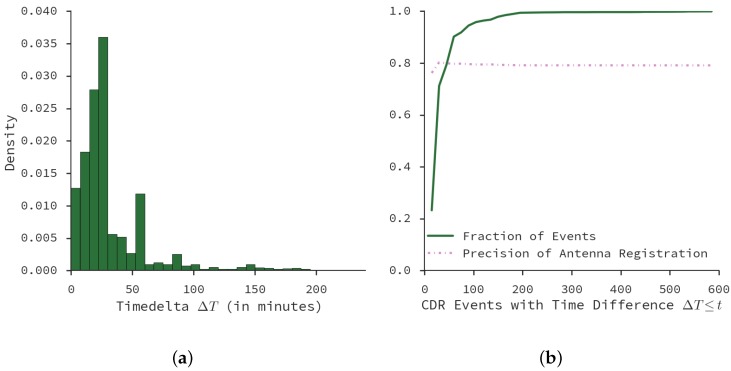
Differences in timing and antenna *id* between a CDR dataset and a network traffic monitoring dataset: (**a**) histogram of timestamp differences, in minutes (capped at 240 min for clarity); (**b**) precision and cumulative distribution of devices considering the timestamp differences.

## Abbreviations

The following abbreviations are used in this manuscript:

AVP: Antenna Virtual Placement
BTS: Base Transceiver Station
CDF: Cumulative Distribution Function
CDR: Call Detail Records
KDE: Kernel Density Estimation
OD: Origin-Destiny
ODS: Origin-Destiny Survey
OSM: OpenStreetMap
PMI: Pointwise Mutual Information
POI: Point of Interest

## Appendix A. Data Events in Call Detail Records

Care must be taken when analyzing data network events. In our dataset these events are triggered every 15 megabytes or every 15 min of active connection, although other operators may have other parameters. Even though data events have been used in the past (for instance, see [10]), due to Internet Protocol being a packet switched protocol, these cut-offs might be arbitrary. This implies, on the one hand, that events may be registered for billing at a later time than their actual timestamp. On the other hand, this implies that the antenna registered for the data event may not correspond to the current antenna the device is connected to. To explore this potential limitation, we briefly analyze an additional dataset: a set of CDR data events from 12 May 2016, as well as a set of network traffic probing measurements, for a random sample of 213 mobile devices. We evaluated the time difference between a CDR event and the last packet received by the corresponding device, as well as the precision of the registered antennas in data events. In both cases, for each data event, we compared its meta-data with the meta-data from the last network package registered. Time differences in registration vary from 0 to 1200 min (μ=35.27, median =25.31). Figure A1a shows the distribution of time differences (left histogram). Figure A1b shows how precision changes when considering the events with lesser or equal time difference than a given threshold. Additionally, it shows the accumulated fraction of mobile devices that are covered when considering only time differences within the threshold. One can see that 71.18% of the events have a time difference lesser or equal than 30 min, and that 90.19% of the events have a time difference lesser or equal than an hour. Previously, analysis of time windows of one hour have been used [4], and thus we find this difference acceptable.

Precision of antenna assignment (μ=0.79, σ=0.01, for all the dataset) has a maximum score of 0.87 when considering events with ΔT≤30 min. The error in antenna assignment may be explained, in part, due that CDR events are collected by the operator, who mantains an up-to-date list of antennas. Conversely, network traffic monitoring is performed by network providers (e.g., Huawei Technologies from Shenzhen, China), who may not have an updated network design.

These results indicate that CDR data events, while not perfect, are good enough to perform our analysis.
